# Investigating effect versus impact in a participatory action research project: Building end-of-life communication competence in residential care facilities for older people

**DOI:** 10.1177/26323524261488419

**Published:** 2026-09-11

**Authors:** Åsa Mikaelsson, Carol Tishelman, Kerrie Noonan, Leo Kowalski, Max Kleijberg, Therese Johansson, Terese Stenfors, Lars E. Eriksson, Ida Goliath

**Affiliations:** 1 Department of Neurobiology, Care Sciences and Society, 27106 Karolinska Institutet, Huddinge, Sweden; 2 Department of Learning, Informatics, Management, and Ethics, 27106 Karolinska Institutet, Stockholm, Sweden; 3 SLSO, Stockholm Health Care Services, Stockholm County Council, Stockholm, Sweden; 4 End-of-Life Care Research Group, Vrije Universiteit Brussel and Ghent University, Brussels, Belgium; 5 58416 Western NSW Local Health District, Dubbo, NSW, Australia; 6 Public Health Palliative Care Unit, La Trobe University, Bundoora Campus, Melbourne, VIC, Australia; 7 Death Literacy Institute, Australia; 8 Department of Health, Innovation and Design, Mälardalen University, Eskilstuna, Sweden; 9 Regional Cancer Centre Stockholm - Gotland, Stockholm, Sweden; 10 Cicely Saunders Institute of Palliative Care, Policy & Rehabilitation, Florence Nightingale Faculty of Nursing, Midwifery & Palliative Care, King’s College London, London, UK; 11 School of Health and Medical Sciences, City St George’s, University of London, London, UK; 12 Stockholm Gerontology Research Center, Stockholm, Sweden

**Keywords:** participatory action research, death literacy, palliative care, end-of-life conversations, mixed methods, impact, advance care planning, survey, residential care homes, GoWish cards

## Abstract

**Background:**

Active involvement of residents, family members and staff in proactive end-of-life conversations remains uncommon in residential care facilities for older people, although these are common settings for the end-of-life. This study derives from a Participatory Action Research project with an intervention aimed at developing Death Literacy among residential care staff, to promote implementation of proactive end-of-life conversations.

**Objectives:**

This article has a two-fold aim: (1) to investigate effects and forms of impact derived from this project intending to improve Death Literacy, and (2) to discuss challenges in this investigation and contribute to critical debate and further inform the use and development of the Death Literacy concept and related index (DLI).

**Methods:**

We combined quantitative analysis of pre-post intervention DLI survey data from staff and qualitative framework analysis of official documents along with transcribed interviews and workshop data from various staff groups. The framework was based on a matrix with Death Literacy features on one axis and forms of impact on the other.

**Results:**

The DLI showed no positive change over time. Effect evaluation was challenged by high baseline mean scores and ceiling effects on the DLI, and issues associated with the use of a participatory research approach, e.g. baseline differences between groups related to recruitment strategies. However, qualitative findings indicated all three forms of impact described in Kleijberg et al.’s model: impact on individual and group development, action-oriented impact, and strategy-oriented impact. These were seen to be related to all Death Literacy features, most notably experiential learning and social action.

**Conclusion:**

Features of Death Literacy were relevant in understanding impact, although the usefulness of the DLI to measure change over time was limited in this study. A matrix framework provided nuanced insight beyond either model alone. Congruence of different methodological assumptions should be carefully considered when designing investigations of effect and impact of complex interventions with limited researcher control.

## Plain language summary

Involving residents and their family members in early conversations about future end-of-life care in residential facilities for older people has several benefits. However, such conversations remain uncommon in elder care. This project was carried out in collaboration with elder care services in a large Swedish city. The research methods used involve doing research ‘with people’ rather than ‘on people’, using flexible processes to promote change. This project aimed at supporting staff by increasing their end-of-life communication competence and helping them gain skills and knowledge—also known as Death Literacy-- needed to engage in end-of-life conversations with residents and their family members. This was done by carrying out a series of workshops with staff groups at three care facilities and studying three comparison facilities that did not participate in workshops. Since this research aims at promoting change, evaluation needed to consider unplanned impacts, and not only direct effects. We used quantitative and qualitative evaluation approaches: surveys before and after the workshop series, and a framework to analyze documents, interviews, and workshop discussions on a matrix, in relation to death literacy and type of impact. We found no positive change over time using the survey data, which was in part related to our participatory methodology. Qualitative data indicated many types of impact, on individual/group development, on accomplishing changes in the workplaces, and on a general strategic level, with changes noted beyond the specific facilities and people involved in the project. Impact was found to be related to Death Literacy features, especially experiential learning and social action. Stakeholder ambitions and desired change should be considered early and systematically, to clarify practice and policy-relevant goals as a basis for evaluation. The features of Death Literacy may be helpful in structuring approaches to raise end-of-life competency.

## Background and aim

The project underlying this article builds further on over 10 years established collaboration in conducting Participatory Action Research (PAR), with the Elderly Services Department of one urban area in Sweden as a key stakeholder. This specific PAR project aimed to implement proactive end-of-life (EoL) conversations in residential care facilities for older people and evaluate the process, effects and impact of doing so. A long-term goal of this initiative was to raise EoL communication competency and Death Literacy (DL)^1^ among elder care staff (see also^2,3^).

The DL concept was developed from a public health perspective to help clarify EoL care competence within a community development framework, emphasizing the transformative potential of engaging in networks of family and friends caring for a person dying at home. DL is defined as the knowledge and skills needed to understand and act upon EoL care options.^1^ Features of DL include *knowledge* (i.e. understanding of palliative care, medications and equipment, policies and EoL care planning processes) and *skills* (i.e. both practical and interpersonal skills such as providing personal care, maintaining relational bonds to the person after death, negotiation about place of death and having conversations with family, friends and professionals about EoL, dying and death). It also includes *experiential learning*, (i.e. the transformative impact of EoL experiences fostering reflection and transformation of behaviors, attitudes and beliefs) and *social action* (i.e. sharing knowledge and skills with outer and inner networks). The Death Literacy Index (DLI) operationalizes the concept.^4^ The use of the DLI as a tool for identifying and evaluating the effect of public health EoL competence-building initiatives has previously been suggested.^5,6^ Although two ongoing intervention programs intend to use the DLI for evaluation^7,8^ no published pre-post evaluation is yet available.

In efforts to decrease the lag between knowledge generation and its uptake, research increasingly seeks to bridge gaps between academic and practice communities by generating new thinking and information about change processes.^9^ PAR shares these aims with more controlled forms of implementation research, and in addition, is consistent with efforts to involve broader groups of relevant stakeholders^10^ in research processes. One ambition of PAR is to conduct research ‘with people’ rather than ‘on people’—referring to all potential users, or benefactors, to develop knowledge of value for stakeholders.^11,12^ PAR is characterized by: 1) partnerships between researchers and other stakeholders, and 2) a dynamic and cyclic process of problem identification, planning, action, and evaluation which incrementally evolves based on lessons learned.^11,12^

However, evaluating PAR projects presents both challenges and potential. One challenge in evaluation thus relates to its focus on not only knowledge generation, but also on achieving change through collaborative processes, i.e. *impact*.^13^ We define impact broadly as (hopefully but not necessarily beneficial) changes that happen in the real world, through processes of knowledge exchange and co-production of knowledge, where new knowledge develops with those who will use and adapt ideas in practice.^14^ PAR evaluation should thus also consider forms of impact that were unplanned and how positive impact can be maximized. This is in stark contrast to evaluating direct *causal effects* on an intervention group versus a comparison group, a form of evaluation that tends to initially focus on controlling the effect of an intervention by limiting its’ impact on other groups. Often in PAR, the action-oriented research approach acts as a catalyst, a stone thrown into the water with impact seen as ripple effects.^15^ Ripple effects can occur at multiple levels; at different stages of PAR; stem from various conditions; and vary in how unintended/unforeseeable and how positive or negative they are. Ripple effects are not just ‘background noise’; their study can generate context-specific understandings and greater insight into the links between participation and impact.^16^ However relatively few studies have examined impact in this manner to date.^17^

From the project onset, we had intended to both test *effects* quantitatively through a pre-post survey and investigate *impact* through qualitative analysis of interviews and workshop data. However, over the course of the project, we increasingly recognized that these ambitions may not be congruent, with underlying methodologies instead appearing to some degree to be in outright conflict. We also noted a lack of harmony between our quantitative results and preliminary analysis of our qualitative data. Given that many share our interest in combining different approaches in efforts to both generate robust knowledge and achieve sustainable change, we hope that presentation and reflection over lessons learned through our attempts can generate new knowledge and critical discussion to further inform both the DL concept and the DLI as a measurement tool, and raise challenges in understanding the effects and impact of PAR studies to help others navigate similar situations. This article therefore has a two-fold aim: 1) to investigate effects and forms of impact derived from our PAR project intending to improve DL, and 2) to discuss challenges we experienced in this investigation.

Research questions addressed here include:

1. What forms, if any, of effect and/or impact can be discerned from this project?
2. What hinders were found in evaluating effects in this PAR project?
3. What potential and difficulties are found in using the DL concept and DLI to evaluate effects and understand impact?

### Study context and motivation

In Sweden, elder care is publicly funded, with local municipalities primarily responsible for delivery of care and support for people aged 65 and older. Older people with extensive care and support needs often spend their EoL in residential care facilities. These include residential care homes with individual apartments for residents and shared spaces on each unit. Care assistants (no formal education needed), assistant nurses (an accredited and protected title in Sweden^18^), and registered nurses are typically present on-site 24/7. Physiotherapists and occupational therapists are generally available during weekdays, while physicians are accessible at scheduled times or via telephone consultation.^19^ Another, less common form of residential care, is assisted living—i.e. facilities with independent living apartments with integrated access to home care services. These were originally intended for older adults who could manage daily life relatively independently. However, as residents typically remain in assisted living facilities until death, their care needs often become increasingly complex over time.^20^ Although 36% of Swedes died in residential care facilities in 2023,^21^ conversations about EoL issues are rare in this context^22–24^ leading to a ‘discourse of silence’.^25^

This discourse of silence is however not limited to elder care in Sweden. An e-survey of over 2000 Swedish residents found low rates of awareness of palliative care in comparison with several other international surveys^26^ with 84% responding they had only some or no awareness. This survey, in line with several others internationally^27^ noted that older age, higher educational levels and being female was significantly related to higher levels of awareness, along with working in a health care setting, or having a family member or friend receive palliative care. Notably, advance care planning is virtually unknown in Sweden, with initiatives being rare exceptions in the country (see e.g. for exceptions Eneslätt et al.^28^) and there is no legal basis for appointing proxy decision-makers or formulating binding advance directives.^29^ One reason contributing to this silence may be the lack of dependence on charitable donations for primarily government-financed EoL care in Sweden, with a potential effect of this being few public information campaigns on this topic.^30^

In this article, we use the term ‘proactive EoL conversations’ to describe structured conversations with residents and/or their family members, focusing on the resident’s values and preferences for (future) EoL care.

These conversations are one component of what is internationally referred to as advance care planning, an umbrella term defined as a process of discussing and documenting EoL care goals and preferences with patients, family members and care providers.^31^ Barriers to implementing proactive EoL conversations in residential care facilities for older people are well-documented and include organizational aspects such as limited time and staffing, lack of experience and training among staff, and unsupportive work cultures.^28,32,33^ Staff who provide daily care for residents are well positioned to initiate proactive EoL conversations.^34^ However, such conversations are often avoided^35^ due to concerns about residents’ capacity to engage^36^ and ethical and emotional considerations – such as fear of causing distress to residents, their family members or due to staff’s own emotional strain.^37^ A whole-system approach has been recommended, integrating EoL communication into routine practice.^38,39^ This includes cultivating a supportive environment, enhancing staff competencies and ensuring access to documentation systems.^40^

## The empirical PAR project: Methods and results

This study, designed as a PAR project, was conducted from January 2022 to May 2025. As previously noted, this PAR project aimed to enhance staff’s EoL communication competency and DL, to support the integration of proactive EoL conversations in three residential care facilities. The conversations were initiated by staff trained as conversation leaders. To enable residents’ active participation, conversations were optimally initiated early in the care trajectory. The DöBra card deck,^41^ a Swedish adaption of the American GoWish card conversation tool,^42^ was used in a series of workshops to train staff as conversation leaders and to support proactive EoL conversations with residents and family members. The DöBra cards have been well studied in a variety of situations, especially among older people in Sweden.^3,29,43,44^ They contain 37 statements addressing physical, practical, existential, and social aspects of EoL.^41^ These statements are intended to be discussed and prioritized in terms of importance to the respondent. We previously found that proactive EoL conversations may promote communication, enable knowledge exchange and foster relationship-building between staff, residents and family members in residential care facilities.^45^

### Recruitment of residential care facilities

In line with PAR principles and building on more than a decade of established collaboration with the research group, one urban municipality’s Elderly Services Department identified three residential care facilities which met researchers’ inclusion criteria to participate in this research project implementing and exploring proactive EoL conversations. Inclusion criteria focused on relative stability among managers and staff, combined with interest and potential to support project engagement. Facilities A, B and C (i.e. implementation group) were invited to participate in the EoL communication competency-building initiative. In addition, care facilities D, E and F were suggested by the Elderly Services Department to act as a comparison group that would not engage with our competency-building initiative and only receive the survey (see below).

### Characteristics of participating care facilities

The implementation facilities were in different, demographically diverse city districts, each with autonomous governance.^20^ The manager at Facility A requested that the two residential care homes and one assisted living facility under her management be included, as they shared goals, management and staff, and were co-located within the same building complex. Facility A thus comprised of these three organizations — one care home with both somatic and dementia units, one with only dementia units and the assisted living facility with no specific profile. Facility B consisted of a building with dementia units, and Facility C included one building with both somatic and dementia units. Each comparison group facility included both dementia and somatic care units. See Table 1 for an overview.

**Table 1.** Overview of participating care facilities: implementation group (Care Facilities A-C) and comparison group (Care Facilities D-F).

​	Implementation group			Comparison group		
	Care facility A^1^	Care facility B	Care facility C	Care facility D	Care facility E	Care facility F
Care orentiation	Dementia units, somatic units and assisted living facility with no specific orientation	Dementia units	Dementia and somatic units	Dementia and somatic units	Dementia and somatic units	Dementia and somatic units
Number of apartments	202	90	85	138	113	88
Number of care staff	208	100	85	138	108	90
Number of managers	5	4	3	5	4	3
​	Staff workshops^2^	​	​	​	​	​
Number of participants	17	9	6	​	​	​
Group composition	A mixed group of care staff, no manager participated.	Group composed of nurse assistants and a manager	A mixed group of care staff, no manager participated.	​	​	​
Appointed implementation facilitator	One participant from staff workshops appointed as implementation facilitator.	A manager participating in staff workshops was appointed implementation facilitator	Shared/unclear facilitator role among managers	​	​	​
​	Manager workshops^2^	​	​	​	​	​
Number of participants	4	2	3	​	​	​
Participants previously trained as palliative care representatives	1	0	1	​	​	​

^1^Facility A consisted of three units.

^2^No workshops in the comparison group.

### Recruitment and composition of staff groups

After recruiting care facilities, we collaborated with managers to purposefully recruit a sample of participants to the series of staff workshops. In line with our participatory approach, managers were encouraged to consider group composition and suggest staff members they perceived as willing and able to support implementation and engage in proactive EoL conversations. This resulted in varying group numbers and composition across facilities, as seen in Table 1. In all groups, some or all participants had previously received a three-day specialized training to be palliative care representatives in their facility and thus were already functioning as resource persons, supporting their colleagues in matters related to palliative care.

### Workshop procedures

As part of the competency-building initiative, two parallel workshop series, one series with staff and one with managers, were conducted and facilitated by researchers (ÅM and IG). Each facility hosted a site-specific series of five, two-hour long staff workshops held over 12 months, to build competence for initiating and implementing proactive EoL conversations.

The format of the workshop series was informed by the research group’s previous research on EoL competency-building in this context.^3,44^ While researchers initially outlined the workshop structure (for detailed information, see Mikaelsson, Eriksson^46^), adjustments were made based on input from staff and managers, in line with PAR. One such modification involved shifting from a train-the-trainer model to direct training of conversation leaders—i.e., preparing staff to initiate proactive EoL conversations themselves. The two first workshops focused on joint reflection using the DöBra conversation tool, i.e. introducing and preparing participants to initiate and facilitate proactive EoL conversations. These sessions included individual and collective reflection about participants’ own EoL care values and preferences using the DöBra cards and applying a research-based, practical guide to support them in engaging in proactive EoL conversations.^47^ Participants first used the cards individually, then with peers or their own family members and subsequently with residents and their family members.

The final three workshop sessions focused on ongoing reflection about experiences of conducting proactive EoL conversations in practice, implementation processes, challenges encountered, and organizational support. In response to staff requests, managers were invited to the fifth staff workshop to engage in discussions about local organizational conditions.

In a parallel process, managers from all implementation facilities were invited to participate in a separate series of four workshops, to exchange knowledge and experiences about the implementation process. Workshops addressed project background and aims, staff workshop content and participant selection, implementation processes and overall reflections.

### Data collection, analysis and results

We combined quantitative and qualitative approaches to investigate effects and impact of the EoL competency-building initiative. Data included survey data collected at two timepoints, publicly available documents, transcripts from staff and manager workshops, and semi-structured interviews with managers and implementation facilitators. To increase readability, we present data collection, analysis and results for the quantitative project component first, followed by that for the qualitative component.

#### The quantitative project component

##### Data collection

**Figure 1.**
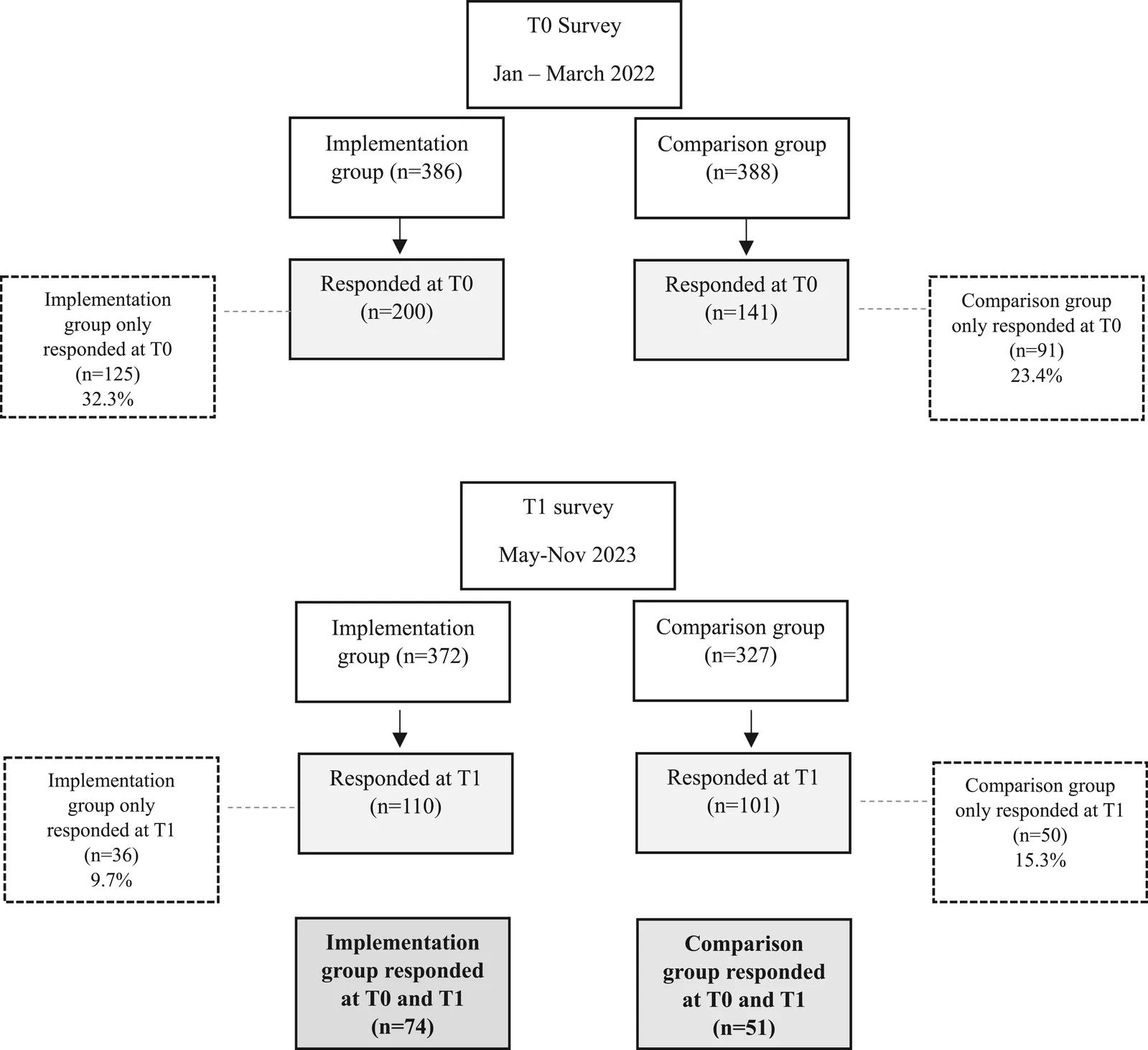
Overview of survey distribution, response rates and repeated participation.

To evaluate the effect of the competency-building initiative, we used a survey including the validated Swedish version of the DLI.^48^ The survey was distributed to all employees across the six facilities on two occasions: at baseline, i.e. prior to project start (T0) and again 16 months after project initiation during the final phase of the workshops (T1). The T0 survey was distributed by email at Facilities A, B, C, D and F, but as requested, in paper format to Facility E, and was open for response from January 17-March 8, 2022. The T1 survey was distributed by email to all facilities and was open from May 5-November 12, 2023. To boost response rates, the window for T1 data collection was extended. Overview of survey distribution, responses and participation are found in Figure 1.

##### Death literacy index

The DLI is a self-assessment instrument comprising 29 items in a total scale, composed of four scales and two subscales: Practical Knowledge comprised of the subscales Talking Support (4 items) and Hands-on Care (4 items), Experiential knowledge (5 items), Factual Knowledge (7 items), and Community Capacity, comprised of the subscales Accessing Help (5 items) and Community Support Groups (4 items).^4^ The items are phrased as statements and rated using a 5-point Likert-type scale, typically ranging from ‘do not agree at all’ to ‘strongly agree’. Scores are transformed to a 0–10 scale, with higher scores indicating higher levels of DL. The DLI has shown satisfactory psychometric properties in its original Australian context^4^ and in adaptations to different languages and contexts^49–51^ with a short form later developed.^52^ In this study we used the Swedish adaption, DLI-S, found to demonstrate strong validity across multiple domains, including content, response processes, and internal structure.^48^

##### Quantitative analyses and results

Due to the distribution of data in the DLI scale and subscales, non-parametric tests were used for all analyses involving these variables.

#### Attrition analysis

We performed attrition analysis to compare participants who responded at both time points and those who only responded at baseline using two independent group t-tests for linear variables and χ^2^ tests for categorical variables. Baseline demographic characteristics of the two groups are presented in Table A1 in the Appendix. Post-hoc analysis found a higher proportion of care assistants responded only at baseline compared with other occupational groups. No other statistically significant differences were found.

Attrition analysis of DLI using Mann-Whitney *U*-tests found statistically significant differences between those responding only at T0 and those responding at both timepoints, such that two-time responders reported higher scores on total DLI and all subscales at baseline, except Community Support Groups (see Appendix, Table A2).

#### Baseline differences between implementation and comparison groups

Table 2 provides demographic data of two-time responders in the implementation group and in the comparison group at baseline. Two independent T-tests for linear variables and χ^2^ tests for categorical variables were used to analyze differences in demographic characteristics between groups. The only statistically significant difference found was that the implementation group had more work experience compared to the comparison group (implementation group mean 17.1 years vs comparison group 11.3 years).

**Table 2.** Baseline demographic characteristics of all two-time responders, comparing implementation and comparison group.

Characteristics	Implementation group (n=74)	Comparison group (n=51)	Comparison statistic^1^
	Mean years (SD)	Mean years (SD)	
**Age**	52.8 (7.5)	51.5 (8.5)	*t* = 0.89 (*p* = 0.37)
**Work Experience in elder care**	17.1 (10.0)	11.3 (9.6)	*t* = 3.30 **(*p* = 0.01)**

​	n (%)	n (%)	​
**Gender**	​	​	*X*^*2*^ = 1.92 (*p* =0.38)
Female	59 (79.7)	45 (88.2)	​
Male	14 (18.9)	6 (11.8)	​
Don’t wish to respond	1 (1.4)	0 (0.0)	​
**Education**	​	​	*X*^*2*^ = 3.04 (*p* =0.80)
Elementary (9 years) or less	5 (6.8)	5 (11.8)	​
High school	25 (33.8)	17 (29.4)	​
Trade school	22 (29.7)	17 (35.3)	​
University (3- years)	8 (10.8)	4 (11.8)	​
Bachelor’s degree (3+ years)	12 (16.2)	6 (7.8)	​
Master’s degree	2 (2.7)	2 (3.9)	​
**Occupation**	​	​	*X*^*2*^ = 0.23 (*p* =0.99)
Care assistant	1 (1.4)	1 (2.0)	​
Assistant nurse	62 (83.8)	42 (80.4)	​
Nurse	5 (6.8)	3 (5.9)	​
Manager	5 (6.8)	4 (11.8)	​
Physical/occupational therapist	1 (1.4)	1 (0.0)	​
**Country/continent of birth**	​	​	*X*^*2*^ = 1.40 (*p* =0.93)
Sweden	22 (29.7)	15 (23.5)	​
Europe outside Sweden	7 (9.5)	5 (11.8)	​
Africa	19 (27.0)	15 (29.4)	​
Asia	21 (28.4)	12 (23.5)	​
South America	4 (5.4)	4 (7.8)	​
**Belief system**	​	​	*X*^*2*^ = 4.57 (*p* =0.206)
There is a God	48 (64.9)	28 (58.8)	​
There is some spiritual power	10 (13.5)	9 (15.7)	​
Don’t know	12 (16.2)	6 (3.9)	​
There is no God or spirit	4 (5.4)	8 (21.6)	​

Statistically significant p-values (p < 0.05) are shown in bold.

^1^Independent two-sample t-test was used to compare continuous variables and χ^2^ was used to examine differences between categorical variables.

Mann-Whitney *U*-tests were used to compare DLI scores of the implementation and comparison groups at baseline. The subscale medians in the implementation group ranged from 7.14 to 10.00, and from 6.88 to 9.50 in the comparison group. No statistically significant differences were found (see Table A3 in Appendix; As DLI is often presented as mean scores, Table A4 presents these to ease comparison.

#### Differences over time

Wilcoxon Signed-Rank Tests were used to compare DLI scores between T0 and T1 for the implementation and comparison groups separately. Sensitivity power analysis indicated that changes between 0.52 and 1.27 on the subscales, were required to exceed the threshold for statistical detectability (i.e., the minimally detectable change). As seen in Table 3, no statistically significant increases in scores were noted over time in either group. In the implementation group, we found, however, a decrease in scores on the DLI subscale Hands-on Care, with median 10.0 at baseline and 8.75 at T1. No other statistically significant changes were noted in either group.

**Table 3.** DLI total and subscales at T0 and T1 within implementation (n=74) and comparison group (n=51).

​	Implementation group (n=74)				Comparison group (n=51)			
Measure	T0 Median	T1 Median	Estimate [95% conf. Int]	W^1^ (*p*-value)	T0 Median	T1 Median	Estimate [95% conf. Int]	W^1^ (*P*-value)
Talking Support	7.50	6.88	0.31 [-0.31, 1. 25]	1286 (0.36)	7.50	6.88	0.63 [-0.31, 1.56]	390 (0.22)
Hands-On Care	10.00	8.75	0.94 [0.31, 1.88]	1276 **(0.001)**	9.38	8.75	0.63 [0.00, 1.25]	376 (0.09)
Experiential knowledge	8.50	8.50	0.25 [-0.50, 1.00]	1017 (0.45)	9.50	9.00	0. 50 [-0.25, 1.00]	508 (0.19)
Factual knowledge	7.14	7.14	-0.36 [-1.07, 0.54]	1115 (0.46)	6.43	7.50	-0.71 [-1.43, 0.18]	450 (0.16)
Accessing Help	7.50	7.25	0.00 [-0.75, 0.75]	1238 (0.86)	7.00	7.00	-0.75 [-1.50, 0.50]	426 (0.21)
Community Support Groups	7.50	7.50	-0.31 [-1.56, 0.94]	984 (0.71)	6.88	7.50	-0.94 [-2.19, 0.31]	300 (0.14)
Total DLI	7.43	7.55	0.14 [-0.40, 0.63]	1475 (0.64)	7.30	7.50	-0.13 [-0.51, 0.26]	604 (0.58)

Statistically significant p-values (p < 0.05) are shown in bold.

^1^Two-sided Wilcoxon signed rank test was used to examine differences over time within the groups.

#### DLI measurement properties

Measurement properties for the DLI and its subscales for those responding at both T0 and T1 were analyzed using Cronbach’s α, ceiling effects analysis, skewness, kurtosis, and Shapiro-Wilk test (see Table 4). Internal consistency was acceptable across subscales (α 0.83 to 0.94), except for Hands-On Care (α = 0.38). We assessed ceiling effects by calculating the proportion of participants scoring within one point of the maximum per subscale. Ceiling effects by subscale ranged from 15.2% (Factual Knowledge) to 56.8% (Hands-on Care). Horney & Tarlov^53^ argue that ceiling effects are problematic if present in ≥15% of participants’ scores. While the properties of the total DLI did not indicate a substantial ceiling effect (3.2%), all subscales evidenced ceiling effects in line with this definition. No floor effects were noted. All subscales were negatively skewed (−0.46 to −1.18), i.e. towards the higher end of distribution. Shapiro-Wilk test indicates that all subscales had a non-normal distribution of data.

**Table 4.** DLI measurement properties; Cronbach’s α, ceiling effect, skewness, and kurtosis at T0 among two-time responders (n=125).

Scale/Subscale	Cronbach’s α	Ceiling Effect (%)^1^	Skewness	Kurtosis	Shapiro-wilk test
Talking Support	0.83	27.2	-0.68	2.81	0.92 (*p* < 0.001)
Hands-On Care	0.38	56.8	-1.02	3.21	0.78 (*p* < 0.001)
Experiential Knowledge	0.92	44.8	-1.18	4.05	0. 84 (*p* < 0.001)
Factual Knowledge	0.87	15.2	-0.49	2.43	0. 95 (*p* < 0.001)
Accessing Help	0.89	16.8	-0.81	2.77	0.91 (*p* < 0.001)
Community Support Groups	0.93	32.0	-0.68	2.35	0.88 (*p* < 0.001)
Total DLI	0.94	3.2	-0.46	2.83	0.96 (*p* = 0.002)

^1^Ceiling effects were assessed as the proportion of participants scoring within one point of the maximum on each subscale.

#### The qualitative project component

##### Qualitative data and analysis

Analyzed data included transcripts from 15 staff workshops, four manager workshops and a total of seven interviews with two implementation facilitators and three managers. All data derived from the implementation facilities A-C.

Furthermore, to investigate more distal impact, official documents in the municipality describing proactive EoL conversations were included in analysis. A search among official documents in the municipality using the term “DöBra cards”, found that they were mentioned in 123 official documents since 2022. Only 12 of these described implementation of proactive EoL conversations and were included in the analysis.

Framework analysis was applied, guided by five steps^54^: familiarization of data through immersion, identifying a framework, indexing data against the framework, summarizing data, and mapping and interpreting across and within the framework. Only data related to this article’s research questions was extracted and indexed in the framework.

ÅM initiated the analysis process by listening to audio recordings and reading transcripts several times. The analysis process was repeatedly discussed and reflected upon by a core group of authors (ÅM, CT, IG, LEE, TS) and findings and conclusions were discussed by all authors.

Kleijberg et al.’s^55^ conceptual model of impact development, based on work from a public health palliative care PAR project, was determined to be relevant for our analysis during the first analytic phase of familiarization with the data. This model describes impact in PAR as both product and process and acknowledges contextual influences in PAR projects. The model identified three forms of impact that are related and evolve from each other^55^ which, while presented separately in the text below, could overlap in our data, as evidenced in Table 5:

1) Impact on individual and group development encompassing: gaining new skills, and changes in thinking and attitudes, including group dynamics
2) Action-oriented impact, including changes in practices of partners, their organizations, and the project group, and
3) Strategy-oriented impact denotes the spread of practice beyond the core group, i.e. either at the same facilities but demanding support from higher organizational levels, or as spread beyond the three facilities that participated in the intervention.^55^

**Table 5.** Matrix including quotes with their source and matrix coding with Forms of impact (x-axis) and features of Death Literacy (y-axis).

​	Quote	Source	Form(s) of impact	Feature(s) of death literacy
1	*I’m thinking that it's difficult [to have proactive EoL conversations]. Is this something that should be brought up fairly soon after they’ve arrived? Or should you wait with it?*	Facility C, Workshop 1	individual/group development	Knowledge
2	*These are important feelings, actually. Strong emotions. Both for me and especially for him [resident]. So, it’s not easy to start talking about death. Especially with older people.*	Facility A, Workshop 2	individual/group development	Experiential learning Skills
3	*How should I think? How should I express myself? Every word can potentially be hurtful or trigger anxiety or something like that.*	Facility C, Workshop 2	Individual/group development	Experiential learning
4	*The challenge here (…) is that it's very multicultural. And it might not be as easy in all countries – maybe there’s not a culture of talking about death at all.*	Assistant manager, Facility C, Workshop 5	Individual/group development	(lack of) Skills
5	*We only have dementia units. In those, it’s really the relatives that you need to follow up with. Most often, these [conversations] are based on the relatives’ thoughts and values and knowledge about the older person. That adds another dimension to the challenge. And then there might not even be any relatives – what do we do then?*	Implementation facilitator, Facility B, Workshop 1	Individual/group development	Other
6	*I’ve said all along that on dementia units, I find it difficult to talk with the dement. (…) They say one thing, and then the next day they forget (…) Right now, on my floor, I don’t think I can have a conversation with anyone*	Facility C, Workshop 5	Individual/group development	(lack of) Skills
7	*You really get a different perspective on things, especially about a topic like this, which can be a bit hard to talk about sometimes. But I think death is a part of life, and (…) it feels natural to talk about it this openly. Putting words to it – that’s not something we always do. But now, it’s been a good opportunity to do that.*	Facility C, Workshop 1	Individual/group development	Skills Experiential learning
8	*Staff have become more confident too. Especially [when it comes to having] this kind of conversation – I used to feel it was very, very shaky for staff with foreign backgrounds…Practice builds skills, and we reflect and talk about our feelings too – how things are, what we think, how they [conversations] are received. (…) It opens doors to further discussions.*	Implementation facilitator, Facility B, Interview 1	Individual/group development Action-oriented	Skills Experiential learning
9	*[Responding to question about what is learned during both workshops and training programs] Conversation techniques (…) And understanding of things like: how much should you listen? How much should you interfere? How much should you interact, and how much can you just be passive and just listen?*	Implementation facilitator, Facility A, Interview 2	Individual/group development	Skills Experiential learning
10	*Yesterday, someone said [during training], ‘This was really good, because it actually makes you think about why you work – when you have to answer these questions.’ (…) [Using the cards] makes you think about how you work, for your own part. To dare to see the person behind the dementia disease.*	Implementation facilitator, Facility A, Interview 1	Individual/group development	Experiential learning
11	*I feel that more people now find it easier to say it – to actually say the word, that she is dead. She’s deceased.*	Implementation facilitator, Facility A, Interview 2	Individual/group development Action-oriented	Skills Experiential learning
12	Participant 1: *I think this [conversation using the DöBra cards] will work better for those who don’t already work so person-centred.* Participant 2: *But really, we already work person-centred. (…). We’ve been having these conversations for a long time, right? It’s just that we didn’t have the cards*	Facility A, Workshop 3	Individual/group development	(lack of) Experiential learning
13	*I’ve found this on all units – people who are proactive, who find it interesting, and who want to learn more.*	Implementation facilitator, Facility A, Interview 2	Individual/group development	Social action – share knowledge and skills with network
14	*I still feel that we’re on the right track, even if it’s going to take time. This is long-term work. (…) I think that planning day we had, there was real engagement. I think everyone needs to talk about these issues in the staff group – to develop their thoughts and reflect on how they themselves think and want things to be. There’s a pent-up need, so that when an older person says, ‘I want to die,’ the response isn’t just, ‘Oh, come now and have a cup of coffee.'*	Manager, Facility A, Manager workshop 4	Individual/group development Action-oriented	Skills Experiential learning
15	*[We’ve had] around ten conversations, and each one has been a bit different. And of course, that was to be expected. But the important thing is to listen – to let the person you’re interviewing talk and explain*	Implementation facilitator, Facility B, Interview 1	Individual/group development Action-oriented	Skills Experiential learning
16	*I think this [project] is really good, but [the conversations are happening] on a very small scale, and it feels like it will take time before it's fully established. (…) It could probably be implemented faster if there were someone really taking the lead and engaging more than we’ve had the opportunity to do here.*	Manager, Facility C, interview	(Lack of) Action-oriented	(Lack of) Social Action
17	*Last year we had… (…) 33 documented conversations (…) We also follow up on these conversations, and I would estimate that we’ve had around fifty conversations between 2022 and 2024, with the majority last year [2024].*	Implementation facilitator, Facility B, Interview 2	Action-oriented impact	Experiential learning
18	*So, I’d say it’s about twenty, maybe twenty-five… [completed EoL conversations…]*	Implementation facilitator, Facility A, Interview 2	Action-oriented impact	Other
19	*[About new internal training held with staff]: Well, I’m not really sure if we’ve included that part [proactive EoL conversations]. We’ve informed the staff about DöBra [ cards] (…) We had a training for the staff, and we did bring up DöBra and a little like that. What’s difficult here is that there are so many different cultures. (…) You can have a conversation, you can talk about it, but many have completely different religions and cultures, which I find makes it hard.*	Manager, Facility C, Interview 1	(Lack of) Individual/group development (Lack of) action-oriented	(lack of) Experiential learning (lack of) Social action
20	*I feel that we managers haven’t fulfilled what we’re supposed to. (…) We haven’t taken the lead on this. We haven’t provided (…) the conditions needed for documentation, for example. (…) [managers] haven’t had any structured follow-up for the conversation facilitators. It’s only been about training all the facilitators (…) But I feel that we haven’t had a forum, and I don’t think we’re there yet.*	Workshop participant and manager, Facility C, Workshop 5	Lack of) Individual/group development (Lack of) action-oriented	Other
21	*Yes, I don’t think my role [as implementation facilitator] is needed anymore [to implement conversations]. Not full-time to that extent.*	Implementation facilitator, Facility A, Interview 2	Action-oriented	Social action
22	*If I disappear from this place, there’s […] no risk that it would just fizzle out, since it’s something we continue with, because we see that it’s good for our residents. It’s good for the organization. It’s good for our staff. (…) It’s a learning process.*	Implementation facilitator, Facility B, Interview 2	Individual/group development Action-oriented Strategy-oriented	Experiential learning Social action
23	*I’m doing a kind of workshop, like the one you held with us. We’ll try laying out the cards. Then we use the documentary. First comes the film. Then we try it out. After that, we go through all the ‘but’, ‘if’, ‘can’t', ‘won’ t, don’t want to’. So. That’s how I structure it. And then we do a round: (…) ‘Who are you thinking of talking to?’ ‘Do you have someone in mind?’ And that’s usually when things (…) start to open up.*	Implementation facilitator, Facility A, Interview 2	Action-oriented	Experiential learning Social action
24	*We’ve also developed this training material after contact with you (…). We’ve changed some, maybe added a little (…). We’ve included some really nice films as well. There’s more content in the actual training now, and yes, a little more reflection and thoughts.*	Implementation facilitator, Facility B, Interview 2	Individual/group development Action-oriented	Experiential learning
25	*With many [of the residents I’ve spoken to], I feel like: ‘I could actually take care of her. Help her feel safe.’ You gain enough [ from the conversations] …*	Implementation facilitator, Facility A, Interview 1	Individual/group development	Knowledge Experiential learning
26	*I had [an EoL conversation] with a person who had really wished that she wouldn’t die alone. […] She had aphasia and was quite advanced in her dementia disease, along with many other sicknesses. So, when she was watched over [at the end of life], and the staff were constantly there, providing palliative care and fulfilling that wish — I, as staff, I felt very satisfied. You’re filled with that feeling of having done something good. […] just then because [we] had had that conversation and were 100% sure that it was what she wanted.*	Facility A, Workshop 5	Individual/group development Action-oriented	Knowledge Experiential learning Social action
27	*We’ve [created a document] for “my priorities” […] It’s divided into ‘personal relationships’, ‘religion and beliefs’, ‘my expectations of care’, and ‘how I want care at the end-of-life”. Then ‘finances’ ended up as its own little tab. (…) On the back, you write the older person’s name, the date it was completed, and who conducted the conversation.*	Implementation facilitator, Facility A, Interview 1	Action-oriented	Social action
28	*We’ve [created a new] form, [and] we document in the medical record (…) that the conversation has taken place and refer to [the form], which is also saved in a binder.*	Implementation facilitator, Facility B, Interview 2	Action-oriented	Social action
29	*We have reflection sessions after a death. With the staff group with the nurse, those who were there, we reflect on how things went. How were the final days? What can we learn? What didn’t go so well? What do we need to work on? And that’s where the [documentation template for the conversations] should also be brought up. Did we fulfil what was said during the DöBra conversation?*	Implementation facilitator, Facility A, Interview 2	Individual/group development Action-oriented	Experiential learning
30	*You need fuel…* *I’ve also seen many times when a project is started and (…) the project leader moves on or quits, or the project ends. Then it can just fizzle out. [That’s why we] have training material. We’ve documented results. We have many people in the organization who can also hold the training.*	Implementation facilitator, Facility B, interview 2	Individual/group development Action-oriented	Social action
31	*We’ve created a list to sign to make sure that [conversations] actually take place, so that we can measure. And that list should be returned to the operations manager.*	Implementation facilitator, Facility A, Interview 2	Action-oriented	Social action
32	*It’s easier to measure hard values. It’s easier to measure [for example] how much toilet paper is used than to measure how much quality is gained from talking to people. (…) You can measure the number of conversations. It’s harder to measure the quality of a conversation…*	Implementation facilitator, Facility A, Interview 1	Individual/group development	Experiential learning
33	*We sit in a group, maybe three people, and then I just lay out a few [cards]. And usually someone starts talking… Then it gets going.* *[Question: So, you’ve done it as a group conversation?]* *Yes (…) some (…) may be further along in their dementia and don’t speak much but talk more easily in a group. (…) They have their buddies they feel safe talking with, and so …*	Implementation facilitator, Facility A, Workshop 5	Action-oriented	Skills
34	*Yes, [we’ve had] group activities [with the DöBra cards], we sit [at the care facility] and talk over a cup of coffee. I wanted to call it ‘qualified death talk’ but wasn’t allowed to.* *They renamed it Ethics Café (…). I think that sounds pretty boring. ‘Qualified death talk’ sounds better, but, well… [laughs]*	Implementation facilitator, Facility A, Interview 2	Action-oriented	Social action
35	*[…] then there was someone else who wanted to join and thought it sounded exciting, so now they have a gym group at Facility A. So, it’s becoming (…) … Yes, there are rings on the water [from the proactive EoL conversations].*	Implementation facilitator, Facility A, interview 2	Action-oriented	Social action
36	*In 2022, the DöBra project was carried out in one unit. The purpose of the project is to strengthen staff so they can better deal with issues related to the end-of-life. The DöBra approach will continue in 2023 and will be implemented across all elder care units.*	Hägersten-Älvsjö Senior Citizens’ Council meeting protocol, 2023	Strategy-oriented	Social action
37	*DöBra – a national research program aimed at developing competence and empowering individuals and staff to better handle issues related to the end-of-life stage – has been implemented, and all units are now using the tool [the DöBra cards] and have teams conducting the conversations.*	Hägersten-Älvsjö City District annual report, 2024	Action-oriented Strategy-oriented	Social action
38	*[In response to the question: how many have you trained?]* *Almost 200, I think (…) in the City district administration. (…) All care homes and home care service and so two… three service houses. (…) Then the home care service has eight units [and] five managers.*	Implementation facilitator, Facility A, Interview 1	Strategy - oriented	Social action
39	*Home care service was also involved in this [training and dissemination] in the beginning (…) But then we realized that it wasn’t enough (…) it was hard to follow up. There needs to be another kind of training (…) because (…) it’s harder to go into someone’s apartment in a regular apartment building, and [harder] for me to follow along without being invited.* *When I’ve been at [residential care homes], I’ve been able to stay with the older person in case something happens (…) I can’t do that in home care.*	Implementation facilitator, Facility A, interview 2	Individual/group development Action-oriented	Experiential learning Social action
40	*I’ve also provided information sessions for other managers in administration, which I created with help from this material — the conversation leader training, and the processes and results. Even suggestions on how other units [in the district] could start this work […] and (…) implement it based on our experiences.*	Implementation facilitator, Facility B, Interview 2	Action oriented Strategy-oriented	Social action
41	*The palliative care representatives group started regular meetings and has begun working on how staff can respond to wishes and questions about end-of-life care. The DöBra card deck has been tested in the group and will be used as a tool in conversations with residents. Licensed staff have received training and have improved their documentation of residents’ wishes for their final stage of life.*	Annual Report 2024 – Elderly Care Department, Kungsholmen	Action-oriented Strategy-oriented	Social action

**Figure 2.**
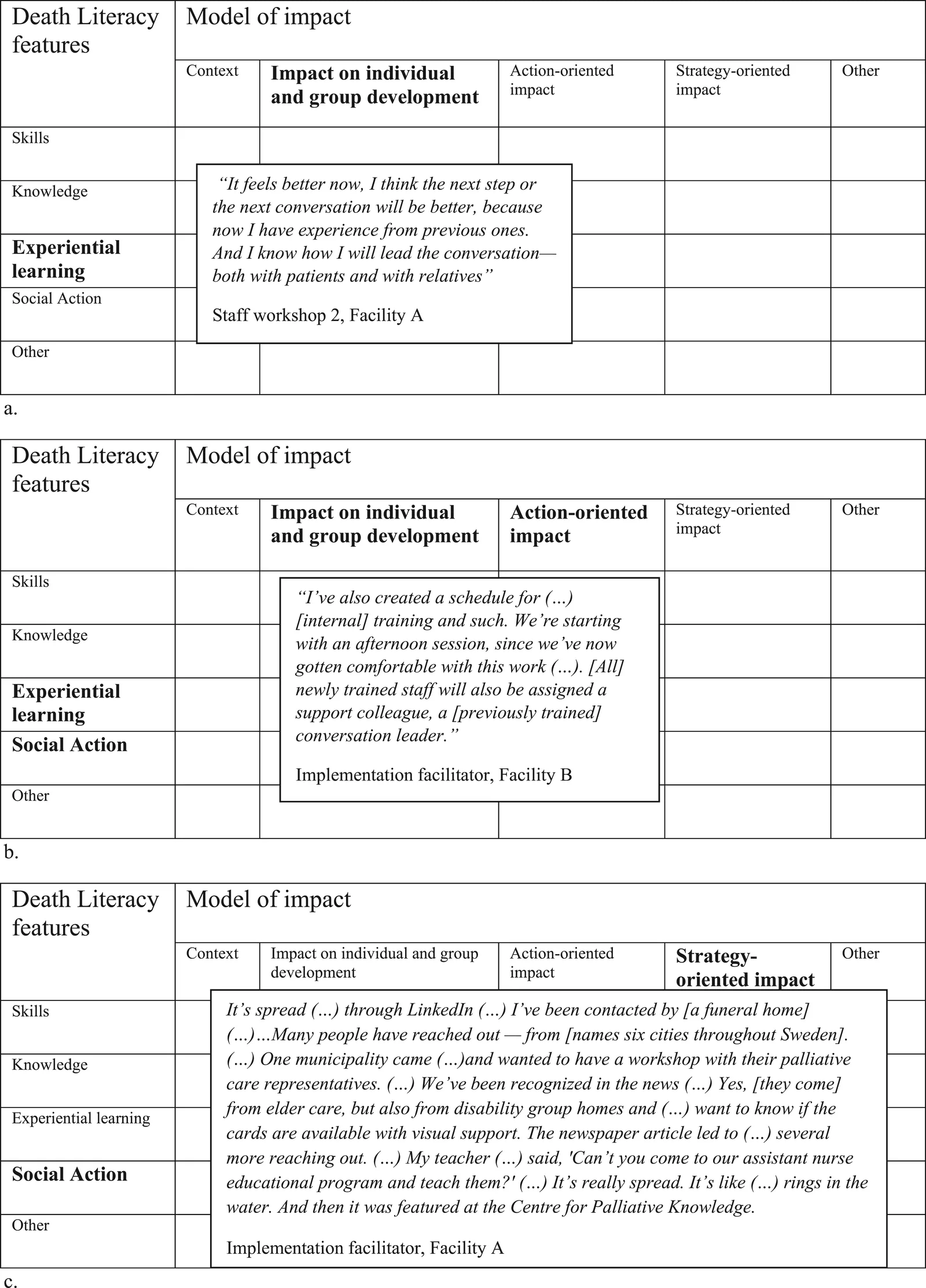
(a)–(c) Matrix for framework analysis with three different examples of data coding. The matrix categories relevant for coding are indicated in larger, bold text, with the quote shown in the matrix.

An initial framework was created using Kleijberg et al.’s^55^ forms of impact as the x-axis and the scales and sub-scales of the DLI as the y-axis.^4^ However, we found limited compatibility between the DLI subscales and the qualitative data and therefore instead used the features of the DL concept as the y-axis of the framework, i.e. Knowledge, Skills, Experiential Learning, and Social Action.^1^ In the framework, we also allowed for data that did not neatly fit into the existing framework categories. Figure 2(a)–(c) provides several illustrations of how quotes were coded to forms of impact and Death Literacy features.

Findings and analytic points are illustrated by numbered quotes found in parentheses in the text, shown in Table 5. Analysis took place in Swedish, with quotes translated to English by two bilingual coauthors. Quotes were shortened, with all efforts made to avoid compromising their meaning. Ellipses (…) indicate omitted words, while square brackets [ ] are used to provide contextual clarification or author comments.

##### Qualitative findings

Findings are presented in relation to the three forms of impact described by Kleijberg et al.^55^

#### Impact on individual and group development

Most data related to this form of impact derives from workshops with staff and interviews with implementation facilitators; little relevant data was noted in manager workshops.

Some participants expressed initial uncertainty about engaging in proactive EoL conversations, citing concerns such as timing (1), the topic’s sensitivity (2, 3), and various communication challenges (4, 5, 6). Talking about dying, death, and EoL care preferences initially felt unfamiliar to some, but putting words to one’s own EoL values was said to aid a shift in perspective, contributing to changes in thinking, emotions and attitudes (7).

This process of learning through doing, sharing, and reflecting—referred to in DL as experiential learning—seemed to shift attitudes and promote preparedness to articulate and engage in proactive EoL conversations (8), even among staff who had previously not done so. Development of these new communication skills took place over time (see Figure 2(a)). The opportunity to build upon experiences and competencies of others was reinforced but also led to new reflections and questions (9).

The use of the DöBra cards during training appeared to support contemplation and encourage a more open and self-reflective approach to some (10). This was said to lead to greater ease in using direct terms such as ‘death’ and ‘dying’ (11). However, in one workshop, two participants questioned the benefit of using the DöBra cards, if already focused on person-centred care (12).

In summary, experiential learning was a strong feature of impact on Individual and group development. This was demonstrated through participants engaging in workshops with collective reflection on personal experiences, values, and priorities using the DöBra cards, and increasingly sharing their own experiences of engaging in proactive EoL conversations. This appeared to support change in participants’ perspectives and reflections on EoL communication. Furthermore, confidence in initiating proactive EoL conversations with residents and families developed, and communication skills such as active listening were fostered. These skills can be related to the DL feature experiential learning primarily in terms of talking support, with transformative effects on beliefs, attitudes and behaviors through learning together.

#### Action-oriented impact

Most data related to action-oriented impact was found in interviews with implementation facilitators and managers, with this form addressed to a lesser extent by staff.

Following the completion of the researcher-facilitated workshops, proactive EoL conversations were initiated in all implementation facilities (13,14, 15, 16), although to different extents (17, 18). While participants were encouraged in workshops to invite colleagues and friends to proactive EoL conversations, some participants went beyond this, taking initiatives to disseminate new knowledge within their own work group. We found the strongest indication of action-oriented impact in data from implementation facilities A and B. In facility C, efforts to improve palliative care were said to continue after the workshop sessions, with staff continuing to be informed about the DöBra cards (19). However, initiating conversations remained challenging in this facility, and this was described as related to unclear roles and lack of organizational support (20). Two years after the workshops started, the implementation facilitators at Facilities A and B described proactive EoL conversations as established practice which were able to continue independent of those initially involved (21, 22). In addition, locally adapted training programs were further developed in these two facilities (23 and Figure 2(b)) to disseminate and apply new knowledge into practice. The content of these built on material and experiences both from the researcher-facilitated workshops and from other resources found through collegial contacts (24).

Engaging in proactive EoL conversations was occasionally described as influencing the quality of the EoL care provided. When staff gained new insights into residents’ values, it was said to help adapt care to better align with these preferences (25), which was particularly relevant when residents experienced communicative and cognitive impairments (26).

Action-oriented impact resulting from participants’ new knowledge was also noted in changes in documenting care preferences to support the new practices, promoting quality care and reflection after a resident’s death. Implementation facilitators developed new documentation templates (27, 28) for this purpose, sometimes assisted by others with more organizational power. Completed templates were stored in residents’ apartments to support care planning and were also used to support team reflection after death to understand the extent to which care had aligned with resident’s values (29).

One implementation facilitator described local training and improved documentation as ‘fuel’ for engagement, aimed at supporting sustainability of their new practice (30). While counting and reporting the number of proactive EoL conversations as a means to measure and visualize implementation progress (31), evaluating quality (32), and perhaps the impact of conversations, was seen as more challenging.

In summary, we found action-oriented impact in descriptions of local initiatives as participants integrated their new knowledge and skills into practice, establishing proactive EoL conversations as routine. Most action-related impact occurred after the workshop series ended and included applying lessons learned to: organize activities using the DöBra cards for residents, revise documentation practices, enhance care planning processes, and establish methods to monitor and report the number of proactive EoL conversations within the organization. Action-oriented impact also included conversation leaders applying their new-found knowledge to provide care in accordance with residents’ preferences, illustrating a feedback loop to individual and group development, as noted in Kleijberg et al.’s^55^ model. Experiential learning invited reflections on local EoL care planning processes and prompted changes in these processes, thereby seeming to be related to the DL feature knowledge. However, other DL features are more pronounced at this impact level, e.g. the DL feature experiential learning, i.e. learning together, transformation of behaviors, and to a lesser extent, the feature skills, related to initiating and conducting proactive EoL conversations. Most predominant here appeared to be the sharing of new knowledge with inner and outer networks, thus related to the DL feature social action.

#### Strategy-oriented impact

Most data related to strategy-oriented impact derives from interviews with implementation facilitators and from official documents from the municipality.

Documents such as patient safety reports, operational reports, and meeting notes from senior citizen councils from the city districts involved in the project, as well as from other city districts, implied that knowledge about the proactive EoL conversation initiatives had been disseminated beyond the initial facilities involved and at higher organizational levels.

However, not all strategy-oriented impact went beyond the original implementation facilities but did demand support at higher organizational levels. One implementation facilitator described how additional activities were initiated at the same facility as a result of the proactive EoL conversations and the insights they generated (33). She described facilitator-initiated group discussions in the dementia units at Facility A, using the DöBra cards for sharing reflections and discussing EoL care issues with residents. The same person later described establishing ‘ethical cafés - serving ‘qualified death-talk’ (34). Another initiative, the establishment of a gym group, was described by this implementation facilitator as a ‘ripple effect’ from the project (35).

Senior citizen councils were informed that local authorities had initiated a process of implementing proactive EoL conversations in all municipal care and support facilities for older people within Facility A’s city district, including home care services and open meeting spaces for older persons (36); this was even documented in this district’s 2024 annual report (37). This was also spoken of by the implementation facilitator (38), responsible for training and follow-up of this city district-wide initiative, who noted challenges in implementing such conversations in home care (39). One implementation facilitator also commented on the vision for dissemination in Facility B’s city district, although it had not yet progressed to the same extent (40). Even one city district in the comparison group documented in their annual report that they had begun a process to use the DöBra cards in proactive EoL conversations (41).

A spinoff project initiated and financed by various stakeholders^1^ after the initial intervention offered further indication of strategy-oriented impact. The short documentary film ‘Finding the way into the conversation’^56^ was developed in a collaboration between a film scholar, film team, project researchers and one implementation facility. Filmed in conjunction with staff workshops in one implementation facility, the documentary was designed as a tool for reflection and inspiration to support engagement in proactive EoL conversations.^57^ According to the implementation facilitator at that facility, the film prompted interest from individuals from across the country – representing diverse organizations and professional backgrounds - who reached out to learn more about proactive EoL conversations and the DöBra cards (see Figure 2(c)). This seemed to have been driven by a combination of factors, including word-of-mouth, media attention and the use of the documentary in various educational contexts. This film was also later used in the local training programs in Facility A and B, mentioned above (25, 26).

In summary, strategy-oriented impact consisted here of both in-house initiatives at implementation facilities with impact on broader organizational practices. Our findings indicate that the dissemination of knowledge about and practice of proactive EoL conversations extended both within and beyond the implementation groups and facilities initially involved, with some uptake observed at higher organizational and policy levels. These forms of dissemination appear linked to the DL feature social action, i.e. through sharing knowledge and skills with inner and outer networks.

## Discussion

To understand research outcomes, we intended to quantitatively examine pre-post scores on the DLI to evaluate potential effects of this PAR project for competency-building with elder care staff in three implementation facilities over time, compared with three comparison facilities. In addition, qualitative analysis of workshop and interview transcripts along with policy documents, was intended to give insight into project impact. While qualitative analysis indicated evidence of the three forms of impact described in Kleijberg et al.’s^55^ model across participating facilities, i.e. impact on individual and group development, action-oriented impact, and strategy-oriented impact, we noted no meaningful change over time using the DLI.

Impact was found to relate to the different features of DL to varying degrees. Although we found limited mention of impact on skills related to personal care of residents, communication skills, a DL feature, were often said to be enhanced as a result of the intervention; this is indeed in line with our original project aims. Findings indicate that the DL feature, experiential learning through reflection about one’s own EoL preferences and shared experiences, facilitated change in beliefs, attitudes and behaviors.^1^ Impact, in the form of ripple effects, was also observed through the dissemination of various new interventions beyond the initial participants, which may be interpreted as aligning with the DL feature social action. Impact on knowledge, the fourth feature of DL, was found to a lesser extent, but some impact seems implied in terms of greater knowledge about EoL care planning processes, leading to reported improvements in carrying out EoL care.

It should be noted that impact was not consistent across all facilities. While some individuals and groups did not seem to change their attitudes or behaviors, this did not seem to be closely related to a particular facility. However, implementation of changes in practice or policy appears closely tied to organizational support, which was more pronounced in two of the three facilities. This highlights the importance of contextual aspects on impact,^55^ including the role of implementation facilitators. It also supports the idea of impact as a process, as noted by Kleijberg et al.^55^; with impact on individual and/or group development as a necessary precursor to action-oriented and strategy-oriented impact, suggesting that without further structural support, sustainable organizational change will not be achieved.

This study has several limitations. There is reason to be cautious about attributing all observed impact, particularly distal strategic forms, solely to this specific project. As noted in the background, this initiative is part of a series of PAR projects conducted over a decade in a well-functioning collaboration with a key stakeholder. It is likely that more distal effects are also related to a trusting and beneficial mutual relationship, proven over time in several PAR projects, in addition to this study.

The lack of a demonstrable effect over time in the quantitative analysis of the DLI could be due to numerous methodological and contextual reasons, including challenges related to lack of control in the PAR design with small, underpowered groups and low response rates, baseline differences among groups, along with ceiling effects in pre-post evaluations due to high baseline scores. A notable limitation of the study is the underpowered sample size. Furthermore, the PAR design encouraged local adaptability, which led to variation, e.g. in composition of intervention sites and workshop groups, leadership strategies, and selection of individual workshop participants and implementation facilitators, as well as some differences in time designated and forms for responding to the DLI. Our collaboration with stakeholders in choosing relatively well-functioning and stable implementation facilities that would promote long-term engagement and sustainability, is likely to have contributed to implementation groups with longer work experience at baseline. While this flexibility aligns with PAR principles, it complicates a controlled pre-post evaluation. These approaches also have differing conceptualizations of researchers’ roles. In controlled quantitative research, a clear separation between the researcher and subject is sought,^58^ whereas the PAR paradigm acknowledges the researcher’s active involvement, participation and influence on the research process.^11^ This suggests that the researchers being known and trusted in the organizations also plays a role in our qualitative findings of impact.

Purposeful choice of intervention sites, and as was the case in our workshops, individual participants, also seemed to have positive aspects. Local ownership and adaptions to context are recognized as essential for the success and sustainability of PAR projects^59^ although they pose challenges to methodological rigor in controlled assessment of effects. Nonetheless, our participatory approach appears to have contributed to both action-oriented and strategy-oriented project impact here. For example, the researcher suggestion of a train-the-trainer model was not initially accepted, but both Facilities A and B later independently developed similar approaches, adapted to their context, with mentorship programs and new educational initiatives run by workshop attendees.

Ceiling effects in DLI subscales were notably high in all groups in our study compared to the only other study in Sweden.^48^ The pronounced ceiling effects represent a limitation, as they reduced the ability to detect change over time. Our study’s exclusive focus on care professionals with EoL care experience may provide one explanation for this, whereas Johansson et al.’s^48^ study included participants from the general population. Few international studies examine DLI ceiling effects. Che et al.’s^49^ validation study from China is an exception; they also found ceiling effects on subscales experiential knowledge and hands-on care, measured on item level in a convenience sample with over 23% health professionals. Recent studies indicate higher levels of DL among both professional and non-professional groups involved in palliative care, e.g. among unpaid carers^60^ and nurses working in palliative care^61^ than in the general population. An ongoing development project found high levels on the DLI among care staff in Australian residential care facilities^7^ after attending an educational intervention, although baseline data are not available for that group. This indicates that ceiling effects are not only found in our study but may be a consideration when using the DLI with other care professionals.

The low Cronbach’s α for the DLI subscale Hands-on care in our study, in addition to the limitations noted above, suggests that the observed negative change over time should be interpreted with caution. This may indicate that the scale does not function well in this population where work tasks are strictly differentiated by staff group; for example, registered nurses administer injections, while assistant nurses often perform tasks such as bathing. In addition, the nature of the intervention itself, with a strong focus on reflection and experiential learning may not lend itself to evaluation through specific and discrete variables. However, the qualitative evaluation suggests that the intervention was helpful in enabling other types of longer-term and more generalized forms of impact.

It should be recognized that the survey sample is biased, by higher attrition among staff with the least education, but who work closely with residents, i.e. care assistants. We also found that those responding to the survey at both baseline and T1 had higher DLI scores from the onset. The generally low response rates in our study may be in part due to practical challenges faced by elder care staff, e.g. a lack of computers, thus further reinforcing the generally lower response rates associated with email surveys compared to paper surveys.^62^ As administration of the survey at T1 inadvertently coincided with the annual employee survey at all intervention and comparison facilities, and staff at Facility A received yet another survey during the same period, survey fatigue may have been present. Response may have been inhibited by staff’s varying levels of verbal and written Swedish language fluency, along with limited control and sense of agency over their time.^63^ The survey contained other instruments in addition to the DLI, as well as demographic questions, and was thus time-consuming. This suggests that this survey approach may not be optimal for obtaining the important perspectives of frontline staff.

We originally chose to use the DLI in the pre-post survey due to its broad scope, encompassing access to, understanding of, and actions related to EoL care. This was considered well in line with the scope and aim of the project. However, it should be noted that DL is based on experiences of engaging in EoL *care ‘in a network of friends and family caring for someone dying at home’,*^1^ whereas this project has focused on the ability to engage in *EoL communication* in an organizational context where EoL care is part of everyday life. In addition to the limitations noted above, the appropriateness of applying the full DLI to measure outcomes in an institutional-like setting may thus be questioned. However, while other instruments for examining EoL competency existed, they were inappropriate for other reasons, e.g. primarily focusing on staff’s psychological preparedness for managing one’s own emotional and existential challenges^64,65^ or directed to other staff groups than those in elder care.^66,67^ Despite the limitations found in using the DLI in this project, it is notable that the more generally-phrased DL features were both relevant and useful in this evaluation. This may suggest that applying a public health perspective to care and services in residential care facilities may add important perspectives for the development of care and support for older people.

## Conclusion

The features of the DL concept provided a valuable analytic tool for qualitative analysis of impact, although its operationalization in the present DLI to measure quantitative effects in terms of change over time in this PAR project in an elder care setting was not useful. There are numerous reasons for the limitations of our analysis, related both to combined use of PAR and an evaluation demanding researcher control, as well as attributes of the DLI itself and the nature of and setting for the intervention. This, along with the limited international data on ceiling effects, suggests that a different, more context-relevant operationalization of the DL features for use in elder care or institutional settings might fill an important gap. On the other hand, we found that Kleijberg et al.’s^55^ model of impact, although empirically developed from a different population in a community-based setting, to be applicable and relevant here. It was also notable that a framework matrix provided more information and analytic precision than using either the DL features or the impact model alone. Congruence of different underlying methodological assumptions needs to be carefully considered when designing ways to investigate both effect and impact of complex interventions conducted in partnership with non-academics.

One specific suggestion resulting from our experiences in this project is the importance of early and systematic consideration of stakeholder ambitions and desired changes, to clarify practice and policy-relevant goals as a basis for evaluation. Features of the DL may also act as sensitizing concepts in planning interventions to raise EoL competency.

Continued systematic and reflective discussion of successful and less successful international efforts in applying conceptual features of the DL and its operationalization in the DLI can contribute to critical debate to further inform the use and development of both.

## Supplemental material

Supplemental Material - Investigating effect versus impact in a participatory action research project: Building end-of-life communication competence in residential care facilities for older people Supplemental Material for Investigating effect versus impact in a participatory action research project: Building end-of-life communication competence in residential care facilities for older people by Åsa Mikaelsson, Carol Tishelman, Kerrie Noonan, Leo Kowalski, Max Kleijberg, Therese Johansson, Terese, Stenfors, Lars E. Eriksson, Ida Goliaths in Palliative Care and Social Practice.

Supplemental Material - Investigating effect versus impact in a participatory action research project: Building end-of-life communication competence in residential care facilities for older people Supplemental Material for Investigating effect versus impact in a participatory action research project: Building end-of-life communication competence in residential care facilities for older people by Åsa Mikaelsson, Carol Tishelman, Kerrie Noonan, Leo Kowalski, Max Kleijberg, Therese Johansson, Terese, Stenfors, Lars E. Eriksson, Ida Goliaths in Palliative Care and Social Practice.

## Data Availability

The datasets are not publicly available due to ethical and confidentiality considerations but are available from the corresponding author upon reasonable request.*

## Note

1 Stakeholders financing the film were: Stockholm City Elderly Services Department, Stockholm Gerontology Research Centre, Region Stockholm and the Centre for Palliative Knowledge in region Stockholm, thus both original project stakeholders and others.
