# Supplementary material for: Investigating effect versus impact in a participatory action research project: Building end-of-life communication competence in residential care facilities for older people
Source: Palliat Care Soc Pract. 2026 Sep 11;20:26323524261488419. doi: 10.1177/26323524261488419 (PMC13570052; doi:10.1177/26323524261488419)
Supplement: Supplementary file 1 [file sj-pdf-1-pcr-10.1177_26323524261488419.pdf]

Table A1. Baseline demographic characteristics of all participants (implementation group and control group) grouped by response pattern; two-time responders and those only responded at T0.

**Characteristics**	**Only responded at T0 (n=216)**	**Two-time responders (n=125)**	**Comparison Statistic (p-value)**[1]
	Mean years (SD)	Mean years (SD)	
**Age**	54.7 (65.5)	52.2 (7.93)	T = -0.55 (p = 0.59)
**Work experience in elder care**	12.9 (10.1)	14.7 (10.2)	T = 1.62 (p = 0.11)
	N (%)	N (%)	
**Gender**			X^2 = 1.12 (p =0.572)
Female	171 (79.2)	104 (83.2)	
Male	44 (20.4)	20 (16.0)	
Don't wish to respond	1 (0.5)	1 (0.8)	
**Education**			X^2 = 1.31 (p =0.97)
Elementary (9 years) or less	21 (9.7)	10 (8.0)	
High school	77 (35.6)	42 (33.6)	
Trade school	62 (28.7)	39 (31.2)	
University (3- years)	19 (8.8)	12 (9.6)	
Bachelor's degree (3+ years)	27 (12.5)	18 (14.4)	
Master's degree	10(4.6)	4 (3.2)	
**Occupation**			X^2 = 13.0 (**p =0.023**)[2]
Care assistant	20 (9.3)	2 (1.6)	
Assistant nurse	163 (75.5)	104 (83.2)	
Nurse	17 (7.9)	8 (6.4)	
Manager	7 (3.2)	9 (7.2)	
Physical/occupational therapist	8 (3.8)	2 (1.6)	
**Continent of birth**			X^2 = 4.77 (p =0.31)
Sweden	49 (22.7)	37 (29.6)	
Europe outside Sweden	35 (16.2)	12 (9.6)	
Africa	63 (29.2)	34 (27.2)	
Asia	48 (22.2)	33 (26.4)	
South America	16 (7.4)	8 (6.4)	
**Belief system**			X^2 = 0.23 (p =0.97)
There is a God	131 (60.6)	76 (60.8)	
There is some spiritual power	36 (16.7)	19 (15.2)	
Don't know	28 (13.0)	18 (14.4)	
There is no God or spirit	21 (9.7)	12 (9.6)	

Statistically significant p-values ($p < 0.05$) are shown in bold.
[1] Independent two-sample t-test was used to compare continuous variables and χ^2 was used to examine differences between categorical variables
[2] Post-hoc results indicated that a greater share of care assistants completed the survey only at T0, compared to participants from other professions.

Table A2. Baseline comparison of DLI scale and subscales between two-time responders and those only responded at T0.

Variable	Median (**Two-time responders**) (n=125)	Median (**Only responded at T0**) (n=216)	Estimate [95% conf. Int]	W[1]	*p*-value
Talking Support	7.50	6.88	0.63 [0.00, 1.25]	15524	**0.020**
Hands-On Care	10	8.75	0.00 [0.00, 0.63]	16248	**< 0.001**
Experiential Knowledge	9	8.00	0.00 [0.00, 0.50]	15342	**0.033**
Factual Knowledge	6.79	5.71	0.71 [0.36, 1.43]	15824	**0.008**
Accessing help	7.50	7.50	0.50 [0.00, 1.50]	15305	**0.039**
Community Support Groups	7.50	7.50	0.00 [0.00, 1.25]	14544	0.230
Total DLI	7.35	6.85	0.60 [0.00, 0.99]	16027	**0.004**

Statistically significant p-values ($p < 0.05$) are shown in bold.
[1] Comparisons were performed using the two-sided Mann–Whitney U test

Table A3. Baseline comparison of DLI scale and subscales of all two-time responders, comparing implementation group and control group

**Variable**	**Implementation group (n=74) Median**	**Control group (n=51) Median**	**Estimate [95% conf. Int]**	**W[1]**	***p*-value**
Talking Support	7.50	7.50	0.00 [-0.63, 0.63)	1868	0.927
Hands-On Care	10.00	9.38	0.00 [0.00, 0.00]	2063	0.340
Experiential knowledge	8.50	9.50	0.00 [-0.5, 0.00]	1816	0.713
Factual knowledge	7.14	6.43	0.357 [-0.357, 1.43]	2114	0.253
Accessing Help	7.50	7.00	0.50 [-0.50, 1.50]	2100	0.284
Community Support Groups	7.50	6.88	0.00 [-0.63, 1.25]	1952	0.741
Total DLI	7.43	7.30	0.11 [-0.48, 0.79]	1968	0.686

[1] Two-sided Mann-Whitney U-tests were used to compare DLI scores of the implementation group and control group at baseline

Table A4. Mean scores of DLI scale and its subscales at T0 and T1 in implementation group and control group

	Implementation group (n=74)		Control group (n=51)	
Variable	Time 0<br>Mean (SD)	Time 1<br>Mean (SD)	Time 0<br>Mean (SD)	Time 1<br>Mean (SD)
Talking Support	6.98 (2.56)	6.71 (2.57)	6.97 (2.60)	6.54 (2.73)
Hands-On Care	9.08 (1.18)	8.21 (2.14)	8.81 (1.44)	8.44 (1.63)
Experiential knowledge	8.23 (1.96)	8.00 (2.14)	8.43 (1.86)	8.10 (1.96)
Factual knowledge	6.84 (2.36)	7.10 (2.09)	6.25 (2.69)	6.67 (2.78)
Accessing Help	6.85 (2.73)	6.84 (2.57)	6.36 (2.73)	6.75 (2.85)
Community Support Groups	6.49 (3.53)	6.72 (2.97)	6.53 (2.91)	7.23 (2.98)
Total DLI	7.44 (1.74)	7.34 (1.68)	7.25 (1.81)	7.31 (1.90)
