# Supplementary material for: Investigating effect versus impact in a participatory action research project: Building end-of-life communication competence in residential care facilities for older people
Source: Palliat Care Soc Pract. 2026 Sep 11;20:26323524261488419. doi: 10.1177/26323524261488419 (PMC13570052; doi:10.1177/26323524261488419)
Supplement: Supplementary file 2 [file sj-pdf-2-pcr-10.1177_26323524261488419.pdf]

Original English-language Death Literacy Index (DLI) with scale and sub-scale headings

1. Practical knowing

1.1. Talking support

PLEASE RATE HOW DIFFICULT OR EASY YOU WOULD FIND THE FOLLOWING TALKING SUPPORT (on a scale of 1-5 between Not at all able to Very able)

1. Talk about death, dying or grieving to a close friend
2. Talk about death, dying or grieving to a child
3. Talk to a newly bereaved person about their loss
4. Talk to a GP about support at home or in their place of care for a dying person

1.2 Hands-on care

PLEASE RATE HOW DIFFICULT OR EASY YOU WOULD FIND THE FOLLOWING HANDS-ON SUPPORT. Undertake the following care duties for the dying (on a scale of 1-5 between *Not at all able* to *Very able*)

5. Feeding a person or assisting them to eat
6. Bathing a person
7. Lifting a person or assisting to transfer them
8. Administering injections

2. Learning from experience

PLEASE RATE HOW MUCH EACH OF THE BELOW STATEMENTS SOUND LIKE YOU (on a scale of 1-5 between Very untrue of me to Very true of me).

My previous experience of grief, loss or other significant life events has

9. Increased my emotional strength to help others with death and dying processes
10. Led me to re-evaluate what is important and not important in life
11. Developed my wisdom and understanding
12. Made me more compassionate toward myself
13. Provided me with skills and strategies when facing similar challenges in the future

3. Existing knowledge

PLEASE RATE HOW MUCH EACH OF THE BELOW STATEMENTS SOUND LIKE YOU (on a scale of 1-5 between Strongly disagree to Strongly agree)

14. I know the law regarding dying at home
15. I feel confident in knowing what documents you need to complete in planning for death

16. I know how to navigate the health care system to support a dying person to receive care
17. I know how to navigate funeral services and options
18. I know how to access palliative care in my area
19. I have sufficient understanding of illness trajectories to make informed decisions around medical treatments available and how that will shape quality of end of life
20. I know about the contribution the cemetery staff can make at end of life

4. Community capacity

<u>4.1 Accessing help</u>

PLEASE RATE YOUR LEVEL OF AGREEMENT WITH THE FOLLOWING STATEMENTS (on a scale of 1-5 between Strongly disagree to Strongly agree).

If I were to provide end of life care for someone, I know people who could help me

21. Access community support
22. Provide day to day care for the dying person
23. Access equipment required for care
24. Access culturally appropriate support
25. Access emotional support for myself

<u>4.2 Support groups</u>

PLEASE RATE YOUR LEVEL OF AGREEMENT WITH THE FOLLOWING STATEMENTS (on a scale of 1-5 between Strongly disagree to Strongly agree).

There are support groups in my community for

26. People with life threatening illnesses
27. People who are dying
28. Carers for people who are dying
29. People who are grieving

Swedish-language Death Literacy Index (DLI-S), with scale and sub-scale headings

1. Praktisk kunskap

1.1. Samtal om att dö, döden eller sorg

Hur beredd skulle du vara att prata om följande?

Ange ditt svar på en skala mellan 1-5, där 1=inte alls beredd och 5=mycket beredd

1. Att prata om att dö, döden eller sorg med en nära vän
2. Att prata om att dö, döden eller sorg med ett barn
3. Att prata med en sörjande person om hens förlust
4. Att prata med vårdpersonal om stöd till en person som kommer att dö i hemmet eller på den plats där hen får vård

1.2. Ge praktisk vård

Hur beredd skulle du vara att göra följande?

Ange ditt svar på en skala mellan 1-5, där 1=inte alls beredd och 5=mycket beredd

5. Att mata en person eller hjälpa hen att äta
6. Att hjälpa en person med att duscha eller att tvätta sig
7. Att lyfta en person eller hjälpa till då en person förflyttas
8. Att ge sprutor

2. Egen kunskap baserad på erfarenhet

Hur stämmer följande för dig?

Ange ditt svar på en skala mellan 1-5, där 1=stämmer inte alls och 5=stämmer mycket väl

Tidigare erfarenheter av sorg, att förlora någon eller andra viktiga händelser i livet har...

9. Gjort mig mer känslomässigt förberedd att ge stöd till andra i processer kring döden
10. Fått mig att fundera på vad som är viktigt och mindre viktigt i livet
11. Gjort mig mer klok och gett mig ny förståelse
12. Ökat min medkänsla för mig själv
13. Gett mig beredskap att klara av liknande utmaningar i framtiden

3. Kunskap om processer och system relaterade till livets slut

Hur stämmer följande för dig?

Ange ditt svar på en skala mellan 1-5, där 1=inte alls och 5=mycket väl

14. Jag känner till de regelsystem som rör dödsfall i hemmet
15. Jag känner till att det finns dokument som kan hjälpa en person att planera inför döden
16. Jag vet tillräckligt mycket om hur vård och omsorg fungerar för att kunna stödja en person att få vård i livets slut
17. Jag känner till processen inför begravning, vart jag kan vända mig och vilka val som finns
18. Jag känner till hur jag får tillgång till palliativ vård i området där jag bor

19. Jag vet tillräckligt för att kunna ta beslut om medicinska behandlingar och förstå hur de kan påverka livskvaliteten i livets slut
20. Jag känner till olika sätt som personal vid begravningsplatser kan vara till hjälp vid begravning

4. Kunskaper om samhället där du bor

4.1. Hur andra kan hjälpa mig med vård i livets slut

Hur stämmer följande för dig?
Ange ditt svar på en skala mellan 1-5, där 1=inte alls och 5=mycket väl

Om jag skulle ta hand om en person i livets slut, så känner jag till var jag kan vända mig för:

21. Att få stöd i området där jag bor, exempelvis från föreningar eller frivilligorganisationer
22. Att få hjälp med att ge en person daglig vård under livets sista tid
23. Att skaffa hjälpmedel som behövs för vård
24. Att få tillgång till stöd som passar personens kultur
25. Att få tillgång till eget känslomässigt stöd

4.2. Stödgrupper

Hur stämmer följande för dig?
Ange ditt svar på en skala mellan 1-5, där 1=inte alls och 5=mycket väl

Jag känner till att det finns stödgrupper för:

26. Personer som har en sjukdom som kan leda till döden
27. Personer som inte har långt kvar att leva
28. Personer som tar hand om någon som är döende
29. Personer som sörjer

Demographic questions in Swedish and English

Demografiska frågor	Demographic Questions
**Hur många år har har du arbetat på din nuvarande arbetsplats?**	**How many years have you worked at your current workplace?**
**Vilken typ av anställning har du? Välj från listan.**<br>• Heltidsanställd<br>• Deltidsanställd<br>• Timanställd	**What type of employment do you have? Choose from the list.**<br>• Full-time employment<br>• Part-time employment<br>• Hourly employment
**Vilken är din yrkesroll på din arbetsplats? Välj från listan.**<br>• Vårdbiträde<br>• Undersköterska<br>• Sjuksköterska<br>• Fysioterapeut/sjukgymnast<br>• Arbetsterapeut<br>• Chef<br>• Annan:	**What is your professional role at your workplace? Choose from the list.**<br>• Care assistant<br>• Assistant nurse<br>• Registered nurse<br>• Physiotherapist<br>• Occupational therapist<br>• Manager<br>• Other:
**Du som är chef, vilken är din yrkesutbildning?**<br>*Denna fråga besvaras endast om du är chef.*<br>• Vårdbiträde<br>• Undersköterska<br>• Sjuksköterska<br>• Arbetsterapeut<br>• Sjukgymnast/fysioterapeut<br>• Om annat, ange:	**If you are a manager, what is your professional education?**<br>*This question is only answered if you are a manager.*<br>• Care assistant<br>• Assistant nurse<br>• Registered nurse<br>• Occupational therapist<br>• Physiotherapist<br>• If other, please specify:
**Vilken är din högsta fullföljda utbildning?**<br>• Grundskola (kortare än 9 år)<br>• Grundskola (9 år)<br>• Gymnasieutbildning<br>• Yrkesutbildning (2 år eller kortare)<br>• Universitets- eller högskoleutbildning kortare än 3 år<br>• Universitets- eller högskoleutbildning 3 år eller mer<br>• Magister/masterexamen<br>• Doktorsexamen	**What is your highest level of completed education?**<br>• Primary school (less than 9 years)<br>• Primary school (9 years)<br>• Upper secondary education<br>• Vocational education (≤ 2 years)<br>• University or college education less than 3 years<br>• University or college education 3 years or more

**Har du för tillfället någon ombudsroll på din arbetsplats?**<br><br>• Ja<br>• Nej<br><br>**Vilken/vilka ombudsroller har du?**<br><br>*Denna fråga besvaras endast om du har en ombudsroll.*<br><br>• Palliativt ombud<br>• BPSD-administratör<br>• Annan ombudsroll, t.ex. språkombud<br><br>**Hur gammal är du?**<br><br>**Vilket kön är du?**<br><br>• Kvinna<br>• Man<br>• Annat<br>• Vill inte svara<br><br>**I vilket land är du född? Välj från listan nedan/annat.**<br><br>**Har du barn?**<br><br>• Ja, jag har barn som bor hos mig<br>• Ja, jag har barn som inte bor hos mig<br>• Nej<br><br>**Har du några av dessa erfarenheter av frågor som rör livets slut? Flera svar är möjliga.**<br><br>• Erfarenhet av familjemedlems, nära släktings eller väns död<br>• Erfarenhet av släktings eller bekants död<br>• Erfarenhet av egen livshotande sjukdom<br>• Erfarenhet av att stödja en person med livshotande sjukdom<br>• Erfarenhet av att vårda en närstående i livets slutskede<br>• Erfarenhet av att stödja en person som sörjer<br>• Arbetslivserfarenhet av att vårda en	• Master's degree<br>• Doctoral degree<br><br>**Do you currently have any representative role at your workplace?**<br><br>• Yes<br>• No<br><br>**Which representative role(s) do you have?**<br>*This question is only answered if you have a representative role.*<br>• Palliative care representative<br>• BPSD administrator<br>• Other representative role, e.g., language representative<br><br>**How old are you?**<br><br>**What is your gender?**<br><br>• Woman<br>• Man<br>• Other<br>• Prefer not to answer<br><br>**In which country were you born? Choose from the list below/other.**<br><br>**Do you have children?**<br><br>• Yes, I have children living with me<br>• Yes, I have children not living with me<br>• No<br><br>**Do you have any of the following experiences related to end-of-life issues? Multiple answers are possible.**<br><br>• Experience of the death of a family member, close relative, or friend<br>• Experience of the death of a relative or acquaintance<br>• Experience of having a life-threatening illness yourself<br>• Experience of supporting a person with a life-threatening illness<br>• Experience of caring for a close person

person i livets slutskede<br>• Jag har ingen erfarenhet av detta<br><br>**Hur länge sedan är det du fick dessa erfarenheter av familjemedlems, nära släktings eller väns död?** *Denna fråga besvaras endast om du har dessa erfarenheter.*<br><br>• Mindre än 2 år sedan<br>• 2 till 5 år sedan<br>• Mer än 5 år sedan<br>• Vet ej/kan ej svara<br><br>**Hur länge sedan är det du fick dessa erfarenheter av släktings eller bekants död?** *Denna fråga besvaras endast om du har dessa erfarenheter.*<br><br>• Mindre än 2 år sedan<br>• 2 till 5 år sedan<br>• Mer än 5 år sedan<br><br>**Hur länge sedan är det du fick dessa erfarenheter av egen livshotande sjukdom?** *Denna fråga besvaras endast om du har dessa erfarenheter.*<br><br>• Mindre än 2 år sedan<br>• 2 till 5 år sedan<br>• Mer än 5 år sedan<br><br>**Hur länge sedan är det du fick dessa erfarenheter av att stödja en person med livshotande sjukdom?** *Denna fråga besvaras endast om du har dessa erfarenheter.*<br><br>• Mindre än 2 år sedan<br>• 2 till 5 år sedan<br>• Mer än 5 år sedan<br>**Hur länge sedan är det du fick dessa erfarenheter av att vårda en närstående i livets slutskede?** *Denna fråga besvaras endast om du har dessa erfarenheter.*<br><br>• Mindre än 2 år sedan<br>• 2 till 5 år sedan<br>• Mer än 5 år sedan	at the end of life<br>• Experience of supporting a person who is grieving<br>• Work experience of caring for a person at the end of life<br>• I have no experience of this<br><br>**How long ago did you have these experiences of the death of a family member, close relative, or friend?**<br>*This question is only answered if you have these experiences.*<br><br>• Less than 2 years ago<br>• 2 to 5 years ago<br>• More than 5 years ago<br>• Don’t know / cannot answer<br><br>**How long ago did you have these experiences of the death of a relative or acquaintance?** *This question is only answered if you have these experiences.*<br><br>• Less than 2 years ago<br>• 2 to 5 years ago<br>• More than 5 years ago<br><br>**How long ago did you have these experiences of having a life-threatening illness yourself?**<br>*This question is only answered if you have these experiences.*<br><br>• Less than 2 years ago<br>• 2 to 5 years ago<br>• More than 5 years ago<br><br>**How long ago did you have these experiences of supporting a person with a life-threatening illness?**<br>*This question is only answered if you have these experiences.*<br><br>• Less than 2 years ago<br>• 2 to 5 years ago<br>• More than 5 years ago<br>**How long ago did you have these experiences of caring for a close person at the end of life?**<br>*This question is only answered if you have these*

Hur länge sedan är det du fick dessa erfarenheter av att stödja en person som sörjer? *Denna fråga besvaras endast om du har dessa erfarenheter.*

- Mindre än 2 år sedan
- 2 till 5 år sedan
- Mer än 5 år sedan

Hur länge sedan är det du fick arbetslivserfarenhet av att vårda en person i livets slutskede? *Denna fråga besvaras endast om du har dessa erfarenheter.*

- Mindre än 2 år sedan
- 2 till 5 år sedan
- Mer än 5 år sedan

Vilket påstående instämmer du mest med?

- Det finns en Gud
- Det finns någon slags andlig kraft
- Jag vet inte riktigt vad jag tror
- Jag tror inte att det finns någon Gud eller andlig kraft

experiences.

- Less than 2 years ago
- 2 to 5 years ago
- More than 5 years ago

How long ago did you have these experiences of supporting a person who is grieving? *This question is only answered if you have these experiences.*

- Less than 2 years ago
- 2 to 5 years ago
- More than 5 years ago

How long ago did you gain work experience of caring for a person at the end of life? *This question is only answered if you have these experiences.*

- Less than 2 years ago
- 2 to 5 years ago
- More than 5 years ago

Which statement do you agree with the most?

- There is a God
- There is some kind of spiritual force
- I am not sure what I believe
- I do not believe that there is a God or any spiritual force
